# Supplementary material for: Isolation and Characterisation of Quercitrin as a Potent Anti-Sickle Cell Anaemia Agent from Alchornea cordifolia
Source: J Clin Med. 2022 Apr 13;11(8):2177. doi: 10.3390/jcm11082177 (PMC9024604; doi:10.3390/jcm11082177)
Supplement: Supplementary file 1 [file jcm-11-02177-s001.zip › Supplementary figures - V3.pdf]

Supplementary figures and tables:

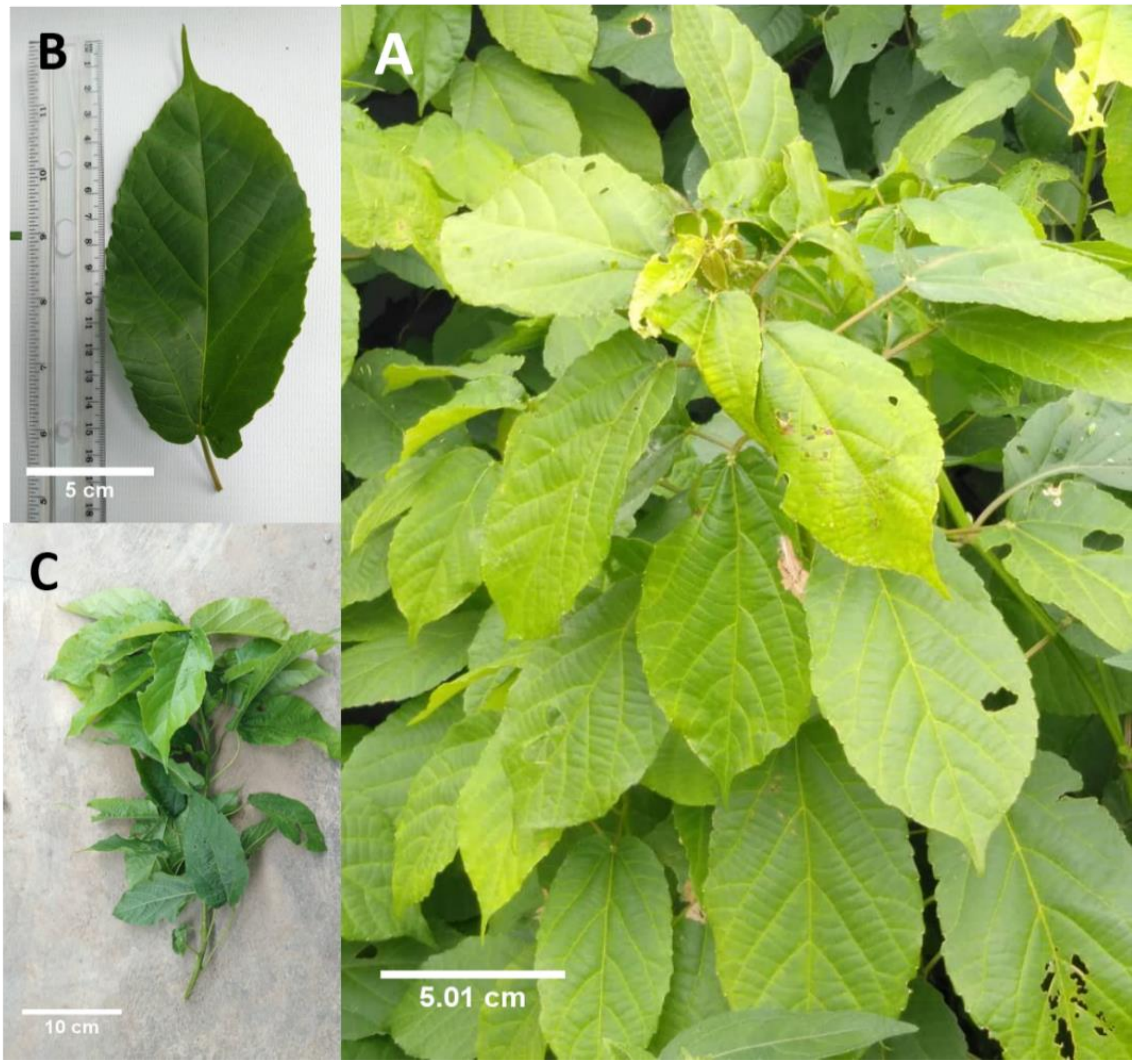

Figure S1. Sample of *Alchornea* used in this study. (A) *in situ*; (B) leaf blade (C) branch. Photographs by Olayemi Adeniyi.

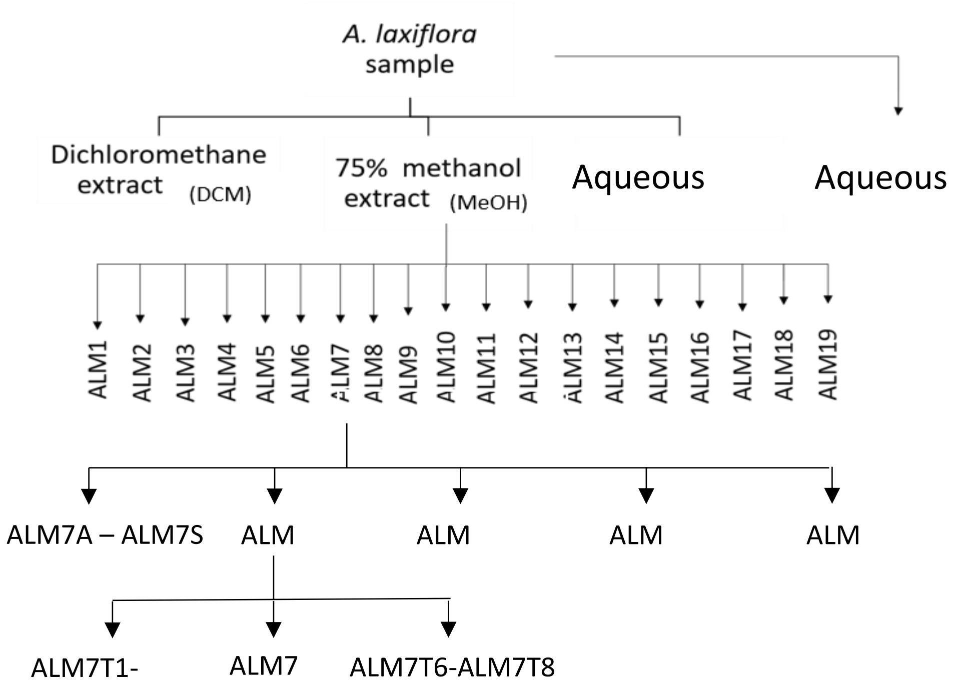

Figure S2. Schematic diagram for the purification of anti-sickling activities in *Alchornea spp.*

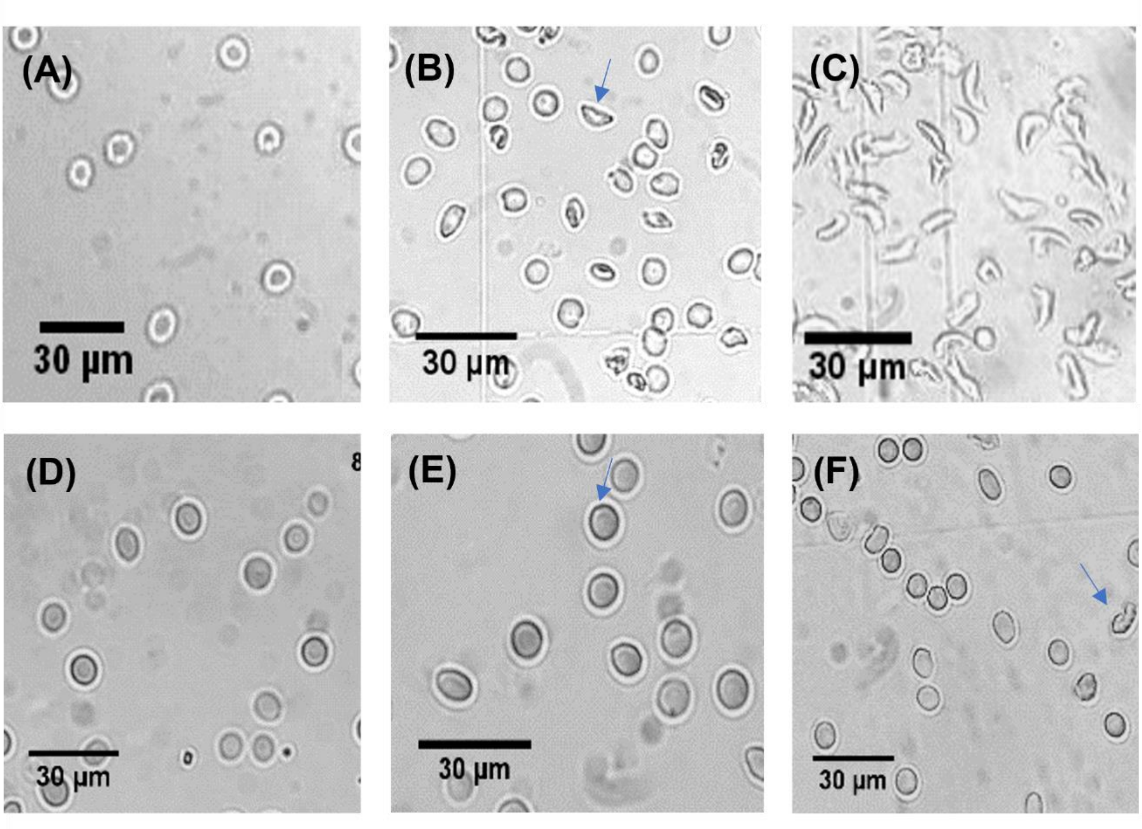

Figire S3 The rffect of *Alchornea spp* extracts and fractions on erythrocyte-sickling in $Na_2S_2O_5$-induced hypoxia**.**

(A) HbAA normoxic **(B)** HbSS normoxic (sickled cell arrowed) **(C)** HbS hypoxic [hyp] (all cells sickled). **(D)** HbSS-hyp+ALM7T5 (0.4 mg/mL)**(E)** HbSS-hyp+ALM7T5 (0.2 mg/mL) **(F)** HbSS-hyp+ALM7T5 (0.05 mg/mL) (abnormal cells arrowed).

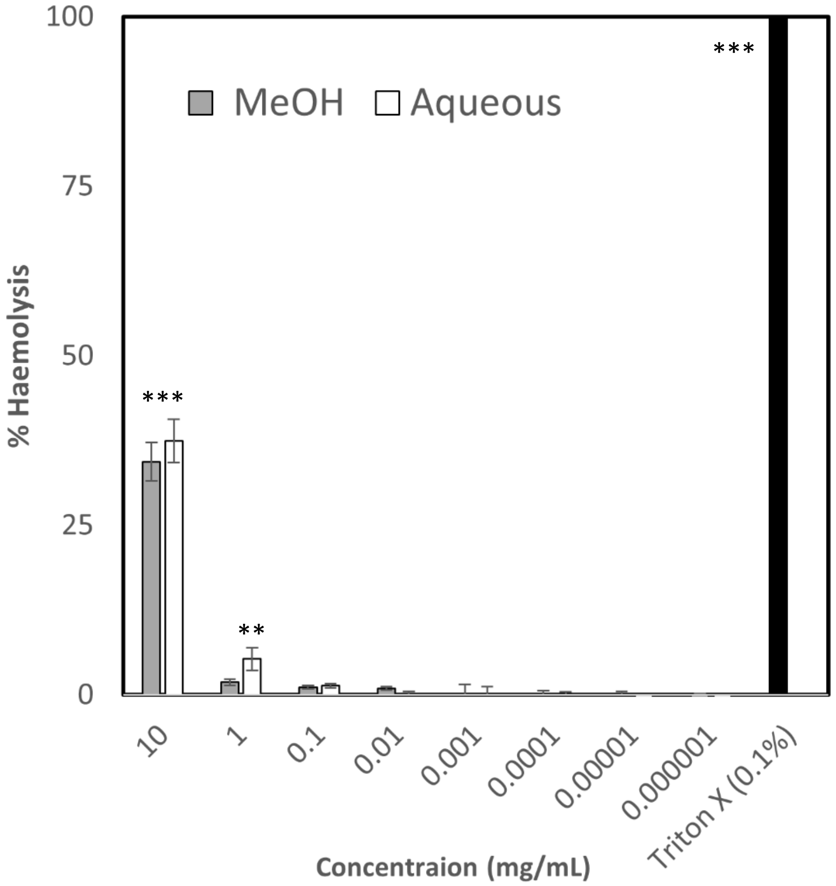

Figure S4. Percentage haemolysis in HbSS RBCs incubated with various concentrations of MeOH and Aqueous extracts of *Alchornea spp* leaves compared with 0.1% Triton X (positive control with 100% haemolysis). Typical data expressed as mean of experiments performed in triplicates. Treatments which show significant ** (P <0.01) and ***(P < 0.001) increases in haemolysis over zero.

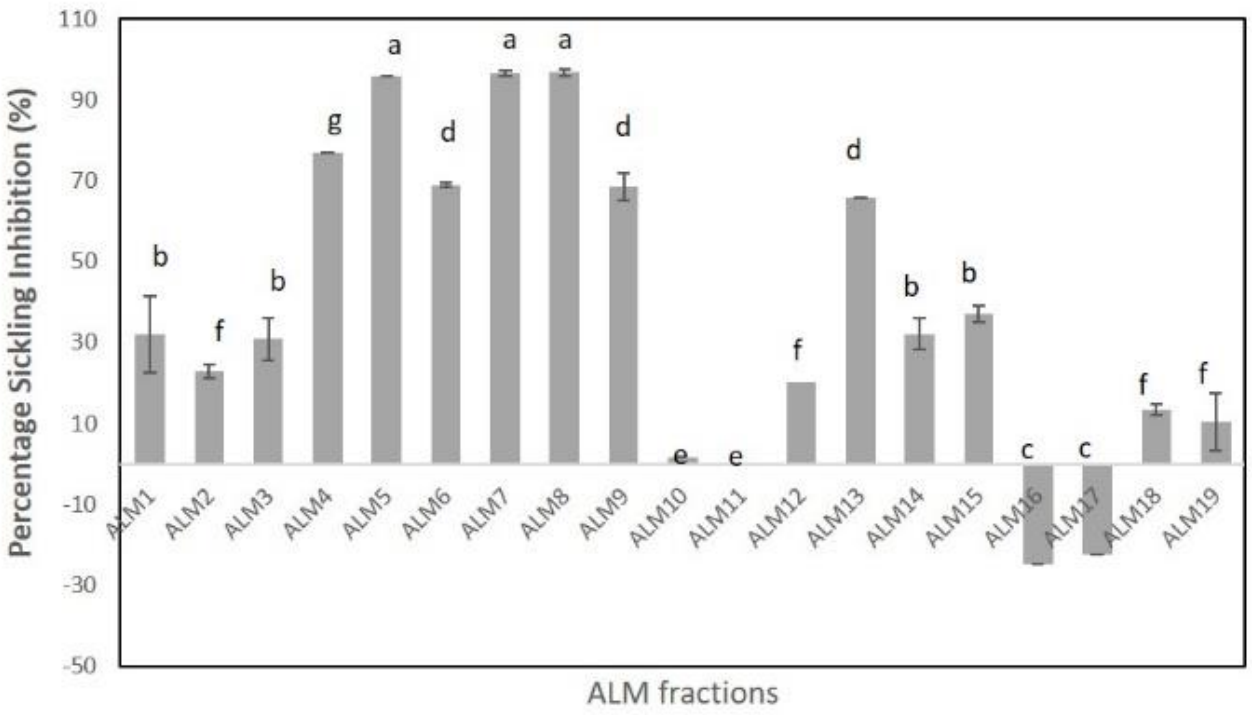

Figure S5. The effect of ALM fractions (1 mg /mL) on erythrocytes-sickling under $Na_2S_2O_5$-induced hypoxic condition.

Groups within which there is no difference are indicated by different letters. The data represents average of 3 similar results from repeat experiments. The letters indicated data sets between between which there was no significant difference.

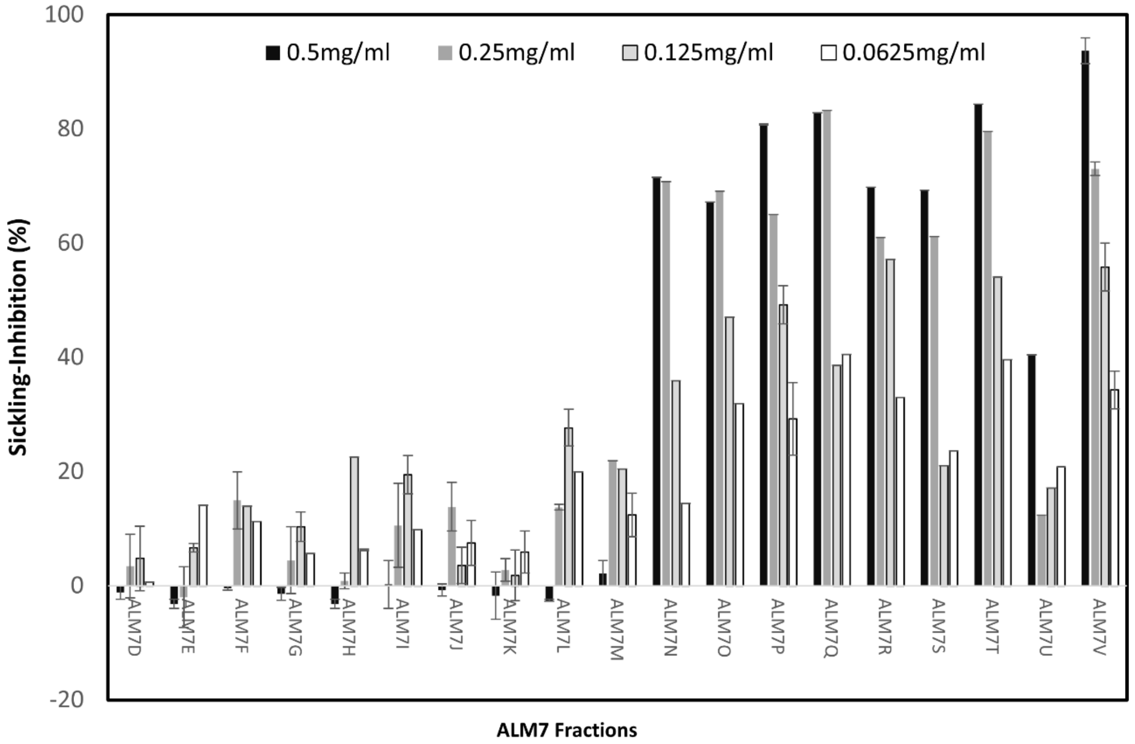

Figure S6. Effect of ALM7 fractions (500 – 62.5 µg/mL) on erythrocytes-sickling under $Na_2S_2O_5$-induced hypoxic condition.

Data represents average of 3 similar results from repeat experiments. Fractions of ALM7N, ALM7O, ALM7P, ALM7Q, ALM7PS, ALM7T, ALM7U and ALM7V showed significantly increases ($P < 0.001$) over zero sickling inhibition activity.

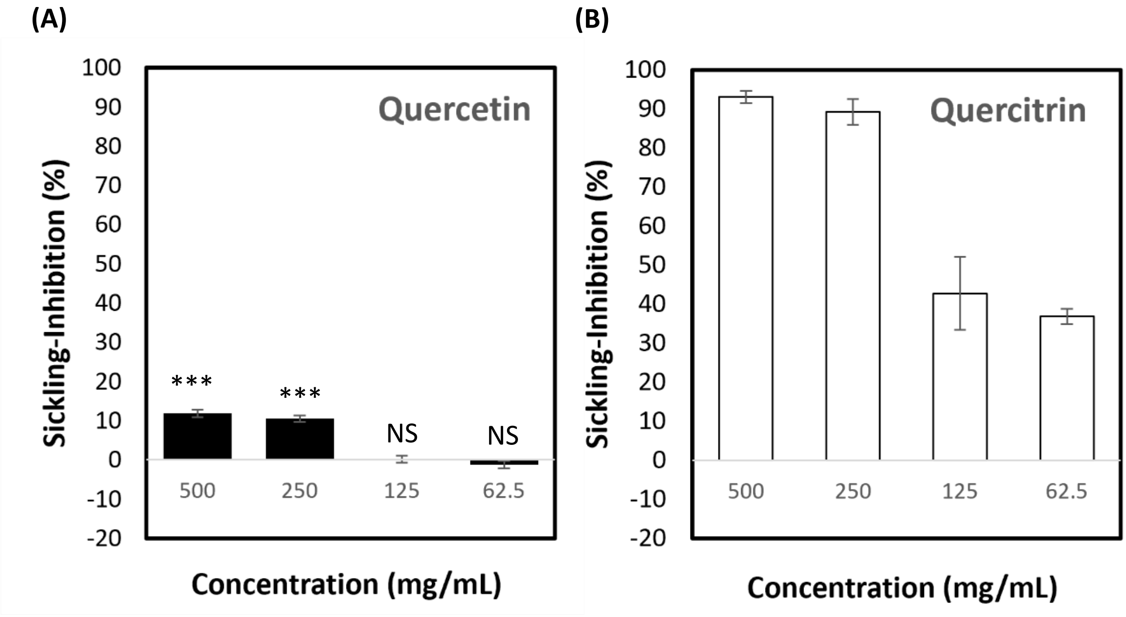

Figure S7. Inhibitory Effects of Quercetin and Quercitrin on HbSS erythrocyte sickling

in vitro assessment of erythrocyte sickling at low oxygen tension induced by $Na_2S_2O_5$ after a 3 hour incubation period with (A) quercetin or (B) quercitrin. Quercetin treatments which show significant increases ($P < 0.001$) over zero sickling inhibition activity is indicated by *** whilst non-significant effects are indicated by NS. All quercitrin treatments showed significantly increases ($P < 0.001$) over zero sickling inhibition activity. These represents data obtained from three typical independent experiments.

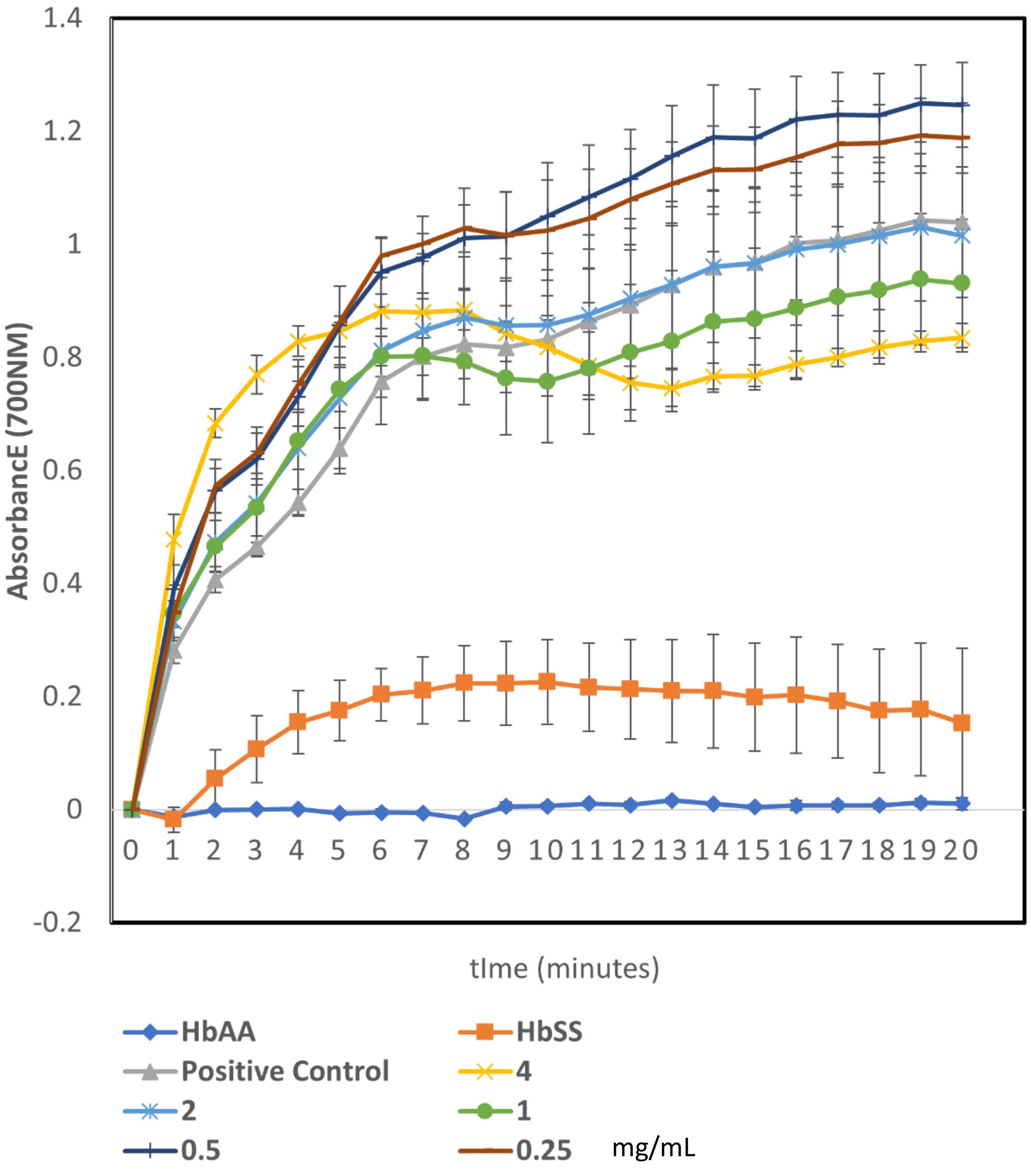

Figure S8. Assessing the potential inhibitory effects of quercetin on HbSS Polymerisation.

Quercetin showed no inhibition of HbS polymerisation with absorbance of polymerised HbS similar to the positive control; deoxyHbS without treatment, at all assayed concentrations. DeoxyHbA (HbAA) was not polymerised as was effectively the negative control. OxyHbS (HbSS) was HbS not subjected to deoxygenation. This represents data obtained from three typical independent experiments performed in quadruples. All data sets were significantly ($P > 0.05$) different to HbAA data after 3 h. All quercetin treated samplesa showed significantly ($P > 0.05$) greater HbSS polymerisation after 1 h.

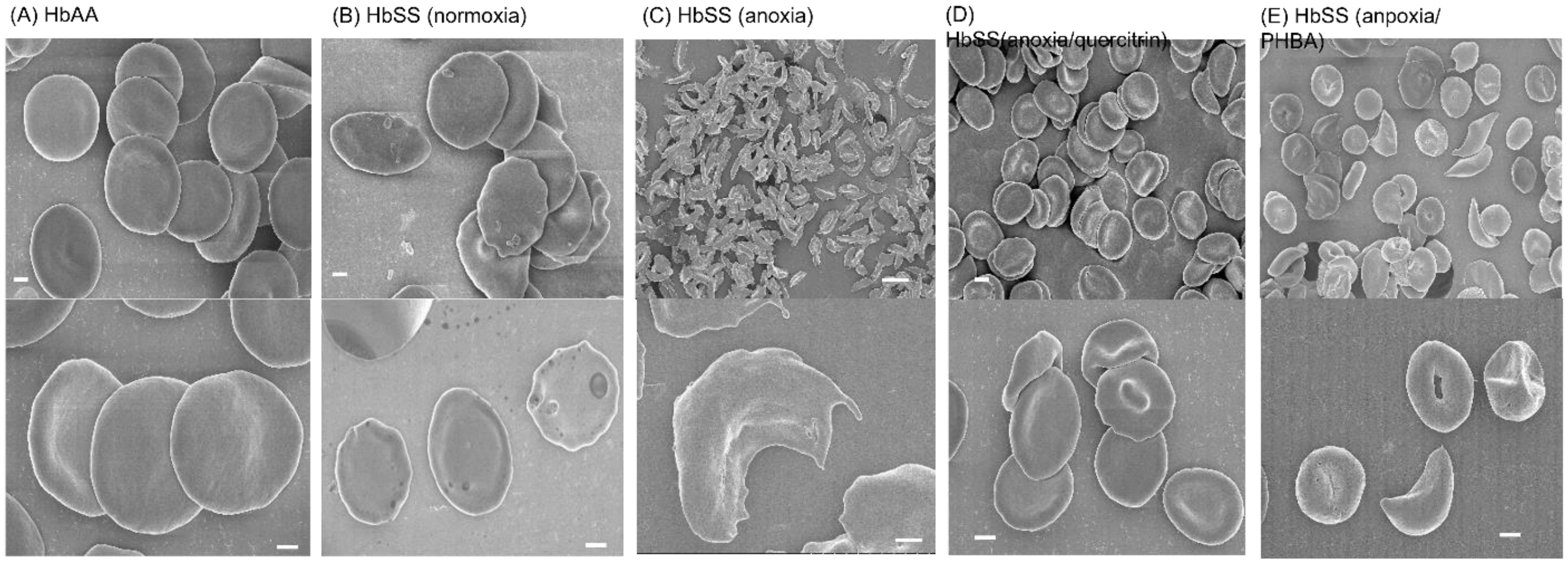

Figure S9. The effect of *Alchornea* spp extracts and fractions on erythrocyte-sickling in N_2- induced hypoxia

Pathway Names	Hits	*P*-value
Ascorbate and aldarate metabolism	4	0.02452
Cysteine and methionine metabolism	8	0.0351
Biosynthesis of unsaturated fatty acids	8	0.0351
Pentose and glucuronate interconversions	7	0.03732
Fatty acid biosynthesis	2	0.04529
Porphyrin metabolism	2	0.04529

Table S2. : Pathway Analysis of *m/z* features significantly ($P<0.05$) different between the treated and untreated groups.
